# The Role of Opportunistic Migration in Cyclic Games

**DOI:** 10.1371/journal.pone.0098190

**Published:** 2014-06-03

**Authors:** Pierre Buesser, Marco Tomassini

**Affiliations:** Faculty of Business and Economics, University of Lausanne, Lausanne, Switzerland; University of Maribor, Slovenia

## Abstract

We study cyclic evolutionary games in a spatial diluted grid environment in which agents strategically interact locally but can also opportunistically move to other positions within a given migration radius. We find that opportunistic migration can inverse the cyclic prevalence between the strategies when the frequency of random imitation is large enough compared to the payoff-driven imitation. At the transition the average size of the patterns diverges and this threatens diversity of strategies.

## Introduction

Cyclic behavior can be observed in evolutionary games when there are more than two strategies available to the players, a well-known case being the *Rock-Scissors-Paper* (RSP) class of games [1]. This behavior is not only of theoretical interest since it is partly responsible for the biodiversity on Earth, and has been actually observed in several biological situations such as the dynamic behavior of side-blotched lizards populations [2], coral reef invertebrates [3], and competition among different bacteria strands [4] among others. These games have been studied extensively both theoretically and by computer simulations. Rigorous results are available for well mixed populations in the infinite population size limit pointing to the fact that the system may converge toward a stable or Lyapunov stable interior rest point, or to an unstable rest point leading to an heteroclinic cycle, depending on the relative values of the payoffs (see, for example, [1], [5], [6]). Cyclic behavior has also been found in studies of the public goods game type when players, besides being able to choose between cooperating or defecting behavior, also have the choice of not taking part in the game (so-called “loner” strategy) [7]. Interestingly, a little later this oscillating behavior was actually observed in an experiment with human subjects by D. Semman et al. [8]. Likewise, in a spatial setting such as two-dimensional grids or, more generally, on relational networks, several results have been obtained. Szabó and Hauert [9] and Szabó and Vukov [10] studied the Prisoner's Dilemma on two-dimensional grids with three strategies: cooperate, defect, and loners and observed that the three strategies survive in a cyclic dominance way akin to the RSP game. A similar phenomenon manifests itself on random graphs but with different characteristics. In [11] Szabó et al. investigated the behavior of the RSP game on regular small-world networks. In more recent work A. Szolnoki and coworkers have further studied the evolutionary Prisoner's Dilemma on spatial grids and random graphs showing that with a third tit-for-tat strategy the system can show a variety of interesting behaviors including stationary and oscillatory states [12]. When agents can only cooperate or defect but have time-dependent learning capabilities Szolnoki et al. [13] showed that cooperator and defectors can coexist and propagating waves appear in the spatially extended system.

In another strand of research players also have the possibility of moving around in space, a feature that is central in ecosystems. Spatial travelling waves and cyclic dominance are typical features of these more biologically realistic settings which are often based on stochastic partial differential equations discretized on a grid to model random diffusion [14], [15]. Another recent paper employs a continuous time space/time formalism in the RSP game with a non-diffusive spatial component [16]. The spatial flux is based on local gradients of relative fitness. In this respect, this study is closer to our approach described below but if focuses on pattern formation and dynamics. Indeed, the strategies are distributed at the start and remain fixed. While the system shows the formation of spirals in space for some initial conditions, and of strategy domains for others, since strategy proportions do not change extinction phenomena are absent. Other important recent works dealing with migration in diluted grid systems are [17], [18].

In this paper we present a new model based on RSP games in which agents enjoy mobility but their displacements are not random; rather, they change place in a purposeful manner. Contingent mobility has previously been used under various forms in two-strategies evolutionary games of the Prisoner's Dilemma, Hawk-Dove, or Stag Hunt types [19]–[24]. The idea here is that the agents possess some basic reactive or elementary reasoning capability that allow them to sense the situation in their local spatial environment and to employ some simple heuristic to move accordingly. Heuristics range from very simple ones such as cooperators moving away from surrounding defectors when the latter are in the majority [19], [23], to more elaborate ones such as “success-driven migration” where agents may try many destinations in space and choose to jump to the most favorable one in terms of expected payoff [20], [24]. Here agents use a simplified form of an heuristic introduced in [24] which consists in randomly trying one single free position in space within a given migration radius and to move there if it is empty and more profitable than the starting one. Our setting requires minimal rational capabilities on the part of the players but it is clearly not adequate for low-level biological organisms such as bacteria where it is likely that movements are almost random. On the other hand, the heuristics used are within the reach of many superior animal populations and certainly of humans. We show in the paper that the addition of opportunistic migration notably changes the dynamical behavior of species. In particular conditions, spatial traveling waves become much longer and tend to diverge with respect to the finite system size causing strategy extinction and thus threatening diversity. On the other hand, in different contexts this result could be seen as a positive one as it tends to stabilize an oscillating system.

## Methods

We investigate a class of two-person, three-strategy, symmetric rock-scissors-paper game as a metaphor for cyclic behavior. These games have the generic payoff matrix 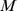 (equation 1) which refers to the payoffs of the row player. The payoff matrix for the column player is simply the transpose 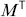 since the game is symmetric.
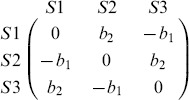
(1)Where 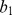 and 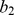 are positive. The set of strategies is 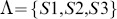.

The Euclidean two-dimensional space is modeled by a discrete square lattice of side 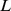 with toroidal borders. Each vertex of the lattice can be occupied by one player or be empty. The *density* is 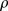 and 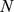 is the number of players. Players can interact with 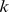 neighbours which lie at an Euclidean distance smaller or equal than a given constant 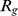. Players can also migrate to empty grid points at a distance smaller than 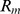. We use three neighborhood sizes with radius 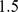, 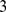, and 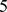; they contain, respectively, 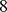, 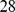, and 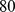 neighbours around the central player.

Each agent 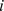 interacts locally with a set of neighbours 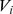 lying closer than 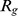. Let 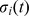 be a vector giving the strategy profile at time 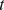 with 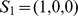, 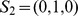, and 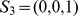, and let 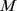 be the payoff matrix of the game (equation 1). The quantity 
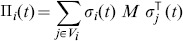
(2)


is the cumulated payoff collected by player 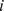 at time step 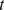.

We use the imitative strategy update called the Fermi rule [5] in which the focal player 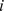 is given the opportunity to imitate a randomly chosen neighbour 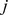 with probability:
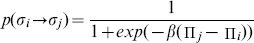
(3)where 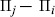 is the difference of the payoffs earned by 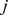 and 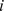 respectively and 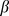 is a constant corresponding to the inverse temperature for the imitation update. When 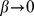 (high temperature) the probability of imitating 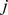 tends to a constant value 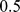 and when 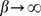 (low temperature) the rule becomes deterministic: 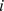 imitates 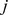 if 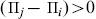, otherwise it doesn't. In between these two extreme cases the probability of imitating neighbour 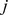 is an increasing function of 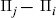.

We use an asynchronous Monte Carlo [5] scheme for strategy update and migration, i.e. players are updated one by one by choosing a random player in each step with uniform probability and with replacement. Then the player migrates with probability 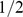, otherwise it updates its strategy.

If the pseudo-random number drawn dictates that 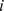 should migrate, then the player considers a randomly chosen position in the disc of radius 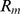 around itself. If the position is already occupied the player does not migrate, otherwise the player computes the payoff that it would obtain in that place with its current strategy. Then player 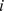 stays at its current position if it obtains higher payoff there, or migrates to the trial position in the opposite case. In order to introduce noise in the migration player 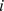 can decide to migrate with probability :
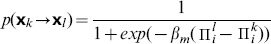
(4)where 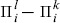 is the difference of the payoffs earned by player 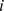 in the positions 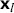 and 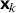, where 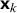 is the original position of player 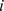 and 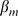 is a constant corresponding to the inverse temperature for the migration. We call these migrations *opportunistic* or *fitness-based*.

We use two measures in order to assess diversity. The first one is called diversity and is simply the normalized product of the strategy frequencies : 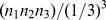. It is proportional to the probability that three randomly chosen players adopt different strategies. Here the highest value of the product is reached when the distribution of the strategies is homogeneous, and if one or more strategy has vanished diversity becomes zero. Indeed, when there are only two strategies remaining, dominance will cause one of the two to disappear afterwards.

The second measure is called the wavelength. It is a rough empirical approximation for the wavelength of a traveling wave or simply for the size of a domain where more than half of the players adopt locally the same strategy. We compute the width of a domain surrounding a player along the 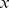 axis 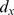 and 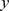 axis 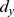 and then choose the shortest width among 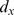 and 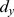 and take the average over all players 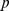. Note that we could obtain similar results by taking the average over 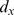 and 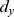. In order to obtain the wavelength around a player 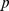 with strategy 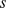 we compute the distance to the border of the 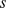 domain along the 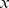 and 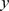 axis in the positive and negative direction around the player 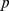. In order to detect if a site 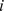 is inside a domain of players adopting strategy 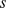, we compute the frequency of players with that strategy inside the Moore neighborhood (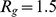) of 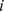, including 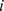. If the frequency is smaller than 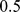, 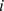 is considered to be out of the domain. Practically we move gradually on the axis until we reach the end of the domain. The next steps take into account the case where the spatial distribution of the population contains empty regions, i.e the frequencies of strategies cannot be computed. In that case, if there are no players in the neighborhood of 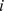, the position of 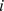 is incremented. Then, if the new place is in a domain with the same strategy we consider that it is still the same domain and continue to increment the test position. Otherwise, the position is considered to be out of the domain and the width of the region without players is subtracted from the total width.

Next, we present here the measure for the invasion speed. We call this measure cyclicity and it takes values 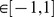. The cyclicity measure for a player at a given time step 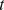 is 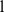 if the strategy has changed according to the natural cycling order (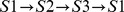) between 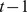 and 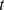, 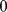 if the strategy has not changed and 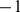 if the strategy changed in the opposite way. The global cyclicity is the average of this quantity over the players during a time interval 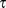 after the system has evolved for 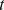 time steps.

For the numerical simulations, the diversity phase-space generated by 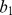 and 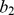 has been sampled with a step of 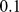 and each value in the phase space reported in the figures is the average of 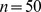 independent runs. For the wavelength plots the number of independent runs is 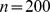. The evolution proceeds by first initializing the population by adding players on grid cells with probability 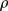. Then the players' strategies are initialized uniformly at random such that each strategy has a fraction of approximately 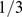. We let the system evolve for a period of 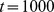 time steps for phase-space diagrams and 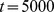 for wavelength plots. In each time step 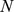 players are chosen for update. We then let the system evolve for 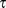 further steps and take the average measure value in this interval. Finally we report the average diversity or wavelength values over the 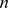 repetitions.

## Results

**Figure 1:**
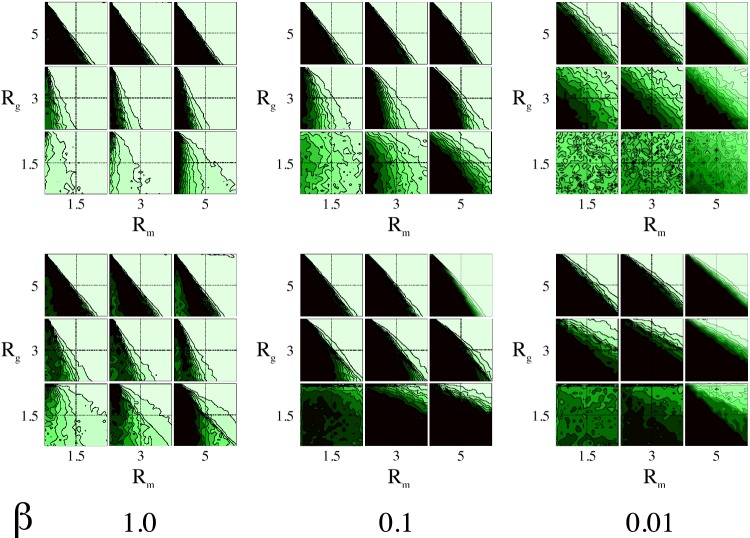
Average diversity levels with random migration (first row) and opportunistic migration (second row) as a function of the game radius 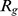 and the migration radius 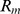. The size of the grid is 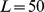 and the density 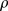 is 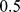. In all cases the initial strategies of the players are attributed uniformly at random. Diversity is maximal for light tones and disappears for black tones as can be seen in the color code bar of Fig. 2.

**Figure 2:**
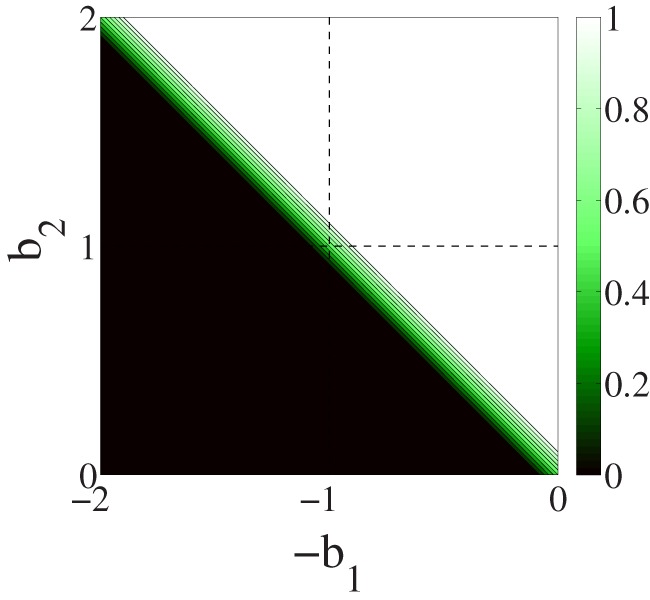
Diversity phase space in a well-mixed population as a function of the game's payoffs 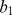 and 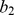 with 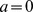. Diversity is maximal for light tones and disappears for black tones.

In order to obtain an overview of the effect of opportunistic migration, the diversity measure is displayed as a function of the game parameters 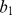 and 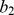 for several values of 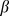. Fig. 1 depicts the diversity phase-space for a lattice of size 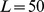 after time 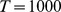 as a function of 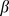, 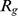 and 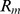. The upper images refer to the random migration case, used here as a benchmark case, and the lower images refer to the opportunistic migration case. By comparing with the well-mixed case shown in Fig. 2, it can be observed that diversity can thrive in adverse games (lower left quadrant) when the interactions radius 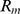 and 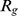 are short (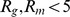). However this does not hold in the opportunistic migration case for all values of 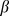 as can be seen in Fig. 1. For 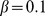 and 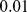 a small game radius 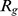 creates the opposite effect for 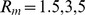 : extinction extend in the upper right quadrant where diversity thrives in the ideal well-mixed case such that nearly all the games of the phase-space lose diversity. For higher game radius 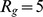 the game space where full diversity thrives is similar to the one found in the random migration case. However this does not imply that the wavelength is similar in the extinction region. Although the small system size used for this exploratory analysis may cause finite-size effects i.e., extinction due to fluctuations, the results show that there is perhaps an interesting phenomenon occurring when 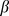 is tuned and thus we try to elucidate it further in the following.

**Figure 3:**
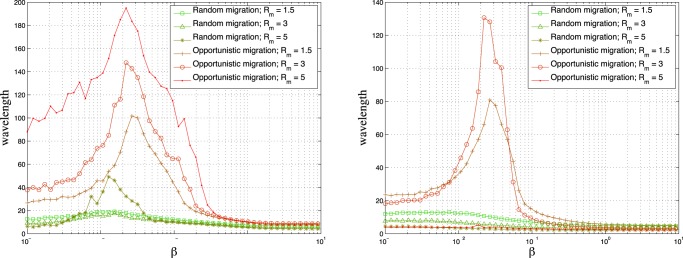
Average wavelength after time 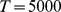 as a function of 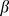 with random migration and opportunistic migration. 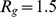
, 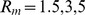. Left image : 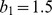, 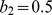 (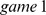). Right image : 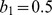, 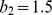 (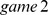). The size of the grid is 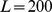 and the density 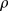 is 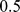. In all cases the initial strategies of the players are randomly attributed.

**Figure 4:**
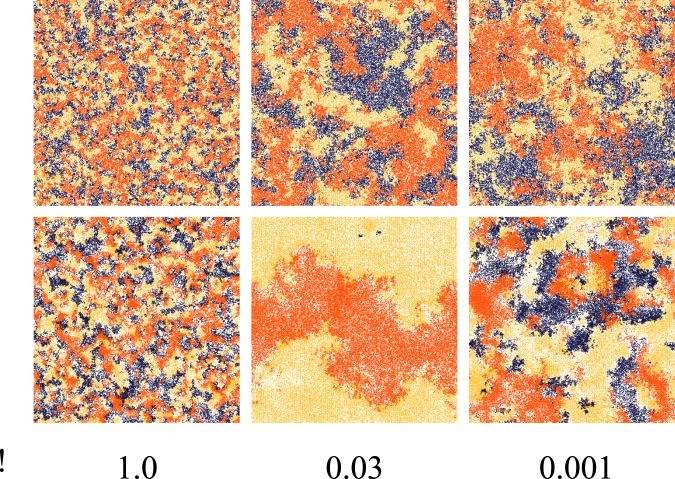
Screenshots with random migration (upper images) compared with opportunistic migration (lower images) as a function of 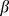, 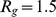, 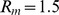, 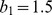, and 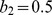 (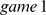). The size of the grid is 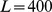 and the density 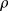 is 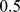. In all cases the initial strategies of the players are randomly attributed. Each color is associated with a different strategy: 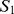 is yellow, 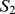 corresponds to blue, and 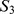 is depicted in orange.

**Figure 5:**
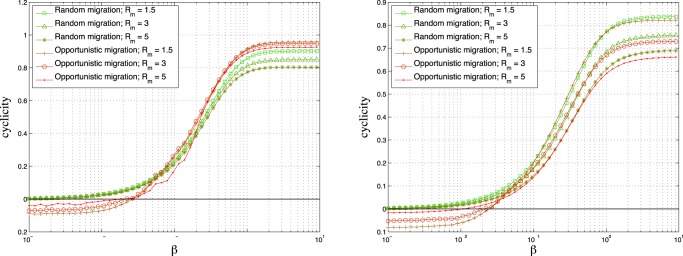
Average cyclicity after time 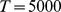 as a function of 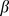 with random and opportunistic migration. 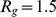
, 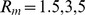. Left image : 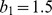, 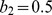 (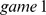). Right image : 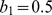, 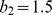 (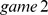). The size of the grid is 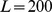 and the density 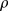 is 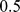. In all cases the initial strategies of the players are randomly attributed.

**Figure 6:**
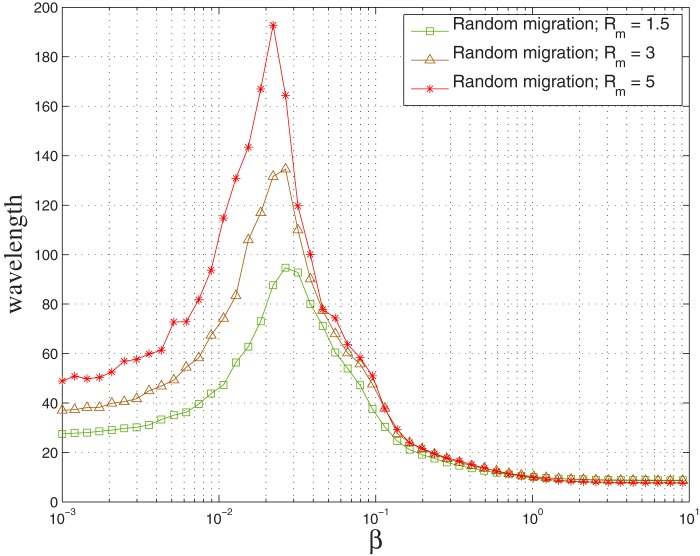
Average wavelength for opportunistic migration after 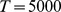 as a function of 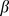 for 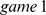: 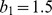, 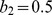. 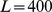
 and 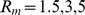. In all cases the density 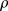 is 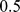 and the initial strategies of the players are randomly attributed.

We study the wavelength on larger lattices as a function of 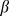 since too small lattices do not let us appreciate large wavelengths due to finite size effects. Since the systematic study of the full game phase space would be computationally too heavy, we report the wavelength for two representative games in the plane. The first game (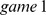) is in the middle of the left lower quadrant of the phase space, 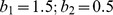, and the second game (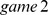) is in the middle of the right upper quadrant, 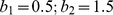. Fig. 3 depicts the wavelength as a function of 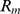 and 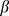 for 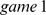 and 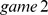, and 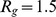, and a frequency of migration of 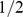. In the opportunistic migration case a marked peak appears for values of 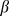 between 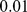 and 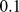. Results for a frequency of migration of 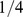 and of 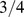 respectively are reported in Material S1. Fig. 4 displays some typical snapshots around the phase transition for random and opportunistic migration. In the central image of the lower row it is clearly visible how domains become larger and extinction sets in for 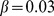 with opportunistic migration. In Fig. 5 the average cyclicity is plotted as a function of 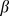 for the opportunistic and random migration cases. It can be seen in the opportunistic migration case that the cyclicity vanishes at the peak and is slightly reversed on the left of the transition so that the position of the peak corresponds to the inversion of the cycling order. This effect can be explained in the extreme case 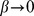 where the imitation tends to be random but the migration is opportunistic. In that case, the players adopting a strategy 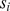 which is payoff-dominated by a strategy 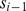 form clusters at the border between the two strategy regions since they try to minimize the number of 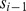 players in their neighborhood. Meanwhile the players adopting the strategy 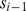 are attracted toward the 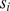 clusters and surround them with a smaller density. Since the strategy update rule is almost random imitation for very small 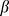 the more clustered players spread their strategy faster than the surrounding players. In fact this effect can be understood in a bipartite population with two degree homogeneous sub-populations 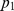 and 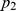 where players imitate randomly their neighbors. A quick calculation shows that the size of the sub-population which has the largest average degree spreads its strategy faster (see Material S1). Also in Material S1it is explained how the effect works using the example of a specific spatial configuration consisting of two neighboring infinite regions with different strategies. In the random migration case it is more difficult to find an explanation since there is no clustering, but the phenomenon is weaker and the peak is less marked. The increase of the wavelength when the cyclicity vanishes is not new and has been studied in [25] in a cyclic voter model with three strategies and a probability to imitate the dominant (dominated) strategy 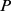 respectively 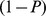 but the phenomenon is not caused by migration, as in our case, since agents don't move and only the 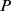 parameter varies. In [26] authors study a spatial five-species predator-prey model with site exchange and invasions between neighbors according to the Rock-Paper-Scissors-Lizard-Spock game. They study the invasion velocities and species density fluctuations as a function of the invasion rates. It is reported that the fluctuations of species frequency diverge and invasion velocities between associations of strategies vanish when tuning the invasion rates. Coming back to the opportunistic migration case, we have checked that the inversion is stable with growing system size. Using short simulation times, such that the system has not reached extinction which means that this data is about the (initial) transient period of the system and not yet at the stationary state, cyclicity can be measured we show that the inversion is similar for all system sizes studied (see figures in Material S1). In Fig. 6 we display the average wavelength for 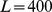, 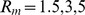 and for 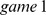: 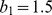, 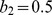. By comparing with the corresponding curve in fig. 3 where 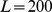 we remark that the peak becomes sharper for 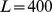 thanks to the larger system size. This is due to the fact that the system can reach extinction before the end of the simulation due to fluctuations of the wavelength even if the mean wavelength is smaller than the system size.

Finally, we study the effect of noise on the migration process using the Fermi rule with parameter 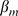 (see Methods section). We observe that, as 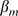 is decreased, the system undergoes a transition inside an interval where the phenomenon gradually disappears. (See Fig. S3 in Material S1). Thus, the global effect of migration noise is to prevent extinction provided that it is high enough, i.e. 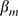 less than 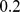. Of course, as migration noise increases, the situation resembles more and more to random walk migration, as it should.

## Discussion

We studied the diversity of strategies in a RSP game in a spatial layout where players migrate opportunistically to more favorable places in their neighborhood. Differently from the many RSP-like systems that have been studied previously in which diffusion is either absent or is random, we found that the diversity is not maintained for large areas of the games' phase space, leading to strategy extinction, when the exponent of the strategy update rule is such that the imitative update is sufficiently noisy. Furthermore, studying the size of the patterns for two representative games as a function of 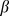 we found that a transition occurs where the size of the patterns diverges and the prevalence of the strategies is reversed. Finally, we also introduced a migration noise and we found that if this noise is larger than a threshold the divergence of the wavelength disappears.

## Supporting Information

Material S1 (PDF)
