# Supplementary material for: The Role of Opportunistic Migration in Cyclic Games
Source: PLoS One. 2014 Jun 3;9(6):e98190. doi: 10.1371/journal.pone.0098190 (PMC4043639; doi:10.1371/journal.pone.0098190)
Supplement: Material S1 — (PDF) [file pone.0098190.s001.pdf]

Supplementary Material

1 Well-mixed population

Suppose that we have two populations (species) p_1 and p_2. All the players from one population have the same type of neighborhood, but the degree of a player is given by the mean number of links to p_1 and p_2. For example players of population p_1 have a degree given by $k_1 = k_{11} + k_{12}$. Where the k_{ij} represent the mean number of link connecting one player of population p_i to players of population p_j. Let N_i be the number of players in p_i, then the variation of N_i corresponding to a particular set of k_{ij} is given by :

$$\dot{N}_i = N_j \frac{k_{ji}}{k_j} - N_i \frac{k_{ij}}{k_i} \tag{1}$$

Now since $N_i k_{ij} = N_j k_{ji}$ is the total number of links between p_1 and p_2 the equation becomes:

$$\dot{N}_i = N_i k_{ij} (\frac{1}{k_j} - \frac{1}{k_i}) \tag{2}$$

Therefore $\dot{N}_i < \dot{N}_j \iff k_i < k_j$. Which means that the population with the highest degree (the players inside the cluster) grows faster in this particular configuration.

2 Configuration Analysis

Suppose that the lattice is composed of two infinite regions of infinite size containing two populations p_1 and p_2 with different densities of players ρ_1 and ρ_2 where the players adopt different strategies, see Fig. 1 where player A belong to the region 1 and player B to region 2 and both have a Moore neighborhood of eight players. We compute the probability that population i grows by one player. In time step t one player is chosen for strategy update. Taking the Moore neighborhood into account we see that only players at the border of the two region can imitate a player from the other region. Since we will compare the probability that p_1 grows with the probability that p_2 grows we can use

$$\frac{\rho_1}{\rho_1 + \rho_2}$$

as the probability that a player of population p_1 at the border is chosen. Since the player chooses a neighbor randomly and imitates it, the probability that population p_1 grows by one is given by

$$\frac{\rho_1 \rho_2}{(\rho_1 + \rho_2)(\rho_2 + \rho_1 5/3)}$$

The same formula is valid for region 2 by exchanging the indexes. Therefore the number of players of type 1 increases faster if and only if $\rho_1 > \rho_2$.

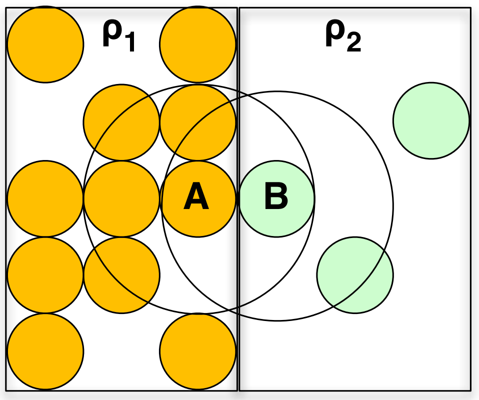

Figure 1:

3 Cyclicity scaling

In Fig. 2 we show the average cyclicity (see main text for the definition) after time $T = 500$ as a function of β for $L = 50, 100, 200, 400$, $R_g = 1.5$, $R_m = 1.5$. Left image : $b1 = -1.5$, $b2 = 0.5$ (*game* 1). Right image : $b1 = -0.5$, $b2 = 1.5$ (*game* 2). In all cases the density ρ is 0.5 and the initial strategies of the players are randomly attributed The inversion of cyclicity is similar in all cases.

4 Migration Noise

Figure 3 shows the dependence of the wavelength on the migration noise parameter β_m for *game* 1 and *game* 2 (see main text).

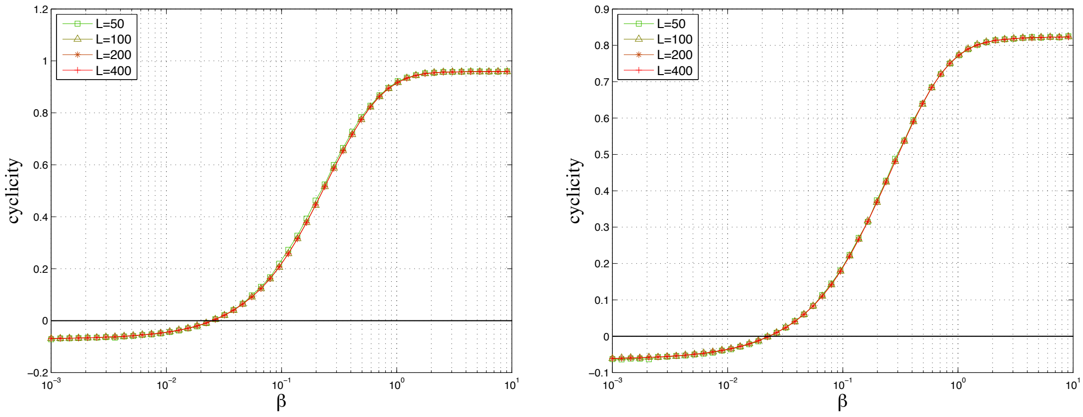

Figure 2:

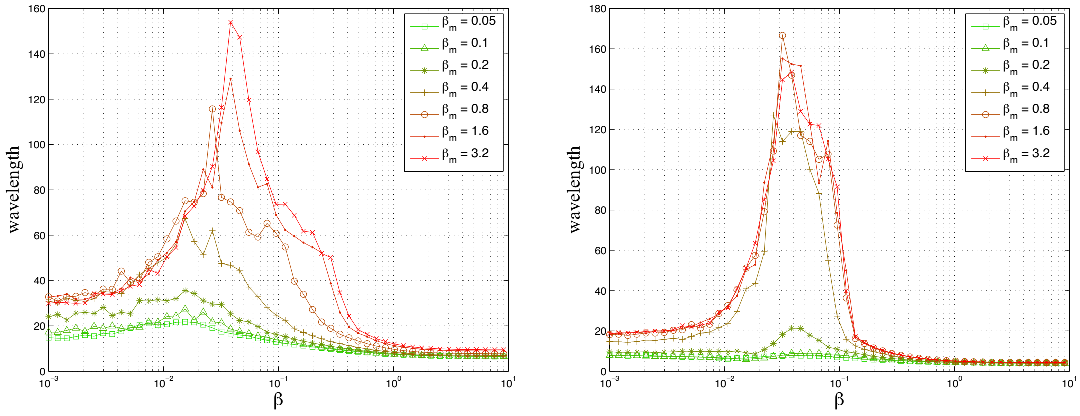

Figure 3: Average wavelength after time $T = 5000$ as a function of β (the exponent of the strategy update rule) for several β_m (the exponent of the noisy migration rule) with opportunistic migration for $R_g = R_m = 1.5$. Left image : $b1 = -1.5$, $b2 = 0.5$ (*game* 1). Right image : $b1 = -0.5$, $b2 = 1.5$ (*game* 2). The size of the grid is $L = 200$ and the density ρ is 0.5. In all cases the initial strategies of the players are randomly attributed.

5 Migration Frequency Dependence

The following figures 4 show the wavelength as a function of β for migration probability 1/4 (left image) and 3/4 (right image). The number of simulation

time steps is 10000 for the system represented in the right image and it is 3333, in order to keep the strategy update number constant to 2500 as in the 1/2 case. In the case of $R_m = 5$ in the right image, the wavelength saturates due to the $L = 200$ system size. To observe the peak, a larger L should be used but this would take an extremely long simulation time.

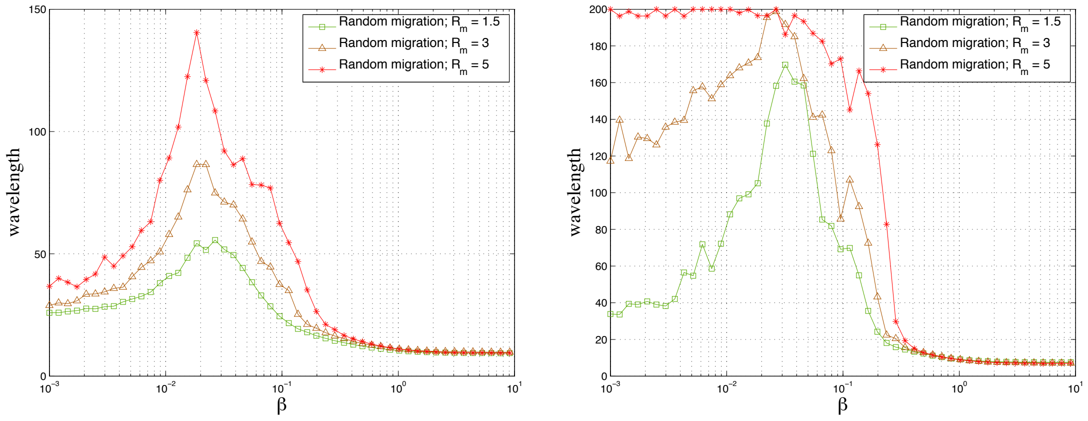

Figure 4: Average wavelength as a function of β (the exponent of the strategy update rule) for several β_m (the exponent of the noisy migration rule) with opportunistic migration for $R_g = R_m = 1.5$, $b1 = -1.5$, $b2 = 0.5$ (*game* 1). The frequency of migration is 3/4 in the right image and it is 1/4 in the left image. The size of the grid is $L = 200$ and the density ρ is 0.5. In all cases the initial strategies of the players are randomly attributed. The saturation of the red curve is due to the wavelength reaching and then overtaking the system size.
